# Supplementary material for: Behavioral control by depolarized and hyperpolarized states of an integrating neuron
Source: eLife. 2021 Nov 5;10:e67723. doi: 10.7554/eLife.67723 (PMC8570696; doi:10.7554/eLife.67723)
Supplement: Supplementary file 1. [file elife-67723-supp3.docx]

**Supplementary File 1a. Table 1. Strain Details**

| **Figures** | **Strain** | **Strain Name** | **Genotype** | **Notes** |
| --- | --- | --- | --- | --- |
| 1-3,  2-S1,  2-S2 | wild-type | CX17882 | *kyEx6329*[pAS03 = *tdc-1p*::nFLP, *elt-2p*::nGFP] | parent of CX0007 |
| N/A | wild-type | CX0007 | CX0007 | Derived from transgene negative controls of CX17882 |
| 1-3,  2-S2 | RIM glu KO | CX17881 | *eat-4(ky1091) eat-4(ky1092)* *III; kyEx6329*[*tdc-1p*::nFLP, *elt-2p*::nGFP] | *eat-4(ky1091) eat-4(ky1092)* backcrossed 3x to N2 See López-Cruz et al., 2019 |
| 1-3,  2-S2 | *tdc-1* | CX17883 | *tdc-1(n3419)* *II; kyEx6329*[*tdc-1p*::nFLP, *elt-2p*::nGFP] | *tdc-1(n3419)* *II* backcrossed 11x to N2 |
| 1-3,  2-S2 | RIM glu KO; *tdc-1* | CX17884 | *tdc-1(n3419)* *II;* *eat-4(ky1091) eat-4(ky1092)* *III; kyEx6329*[*tdc-1p*::nFLP, *elt-2p*::nGFP] | *tdc-1(n3419)* *II* backcrossed 11x to N2 *eat-4(ky1091) eat-4(ky1092)* backcrossed 3x to N2 See López-Cruz et al., 2019 |
| 2-S1 | *elt-2p*::nGFP; wild-type | CX18118 | *kyEx6458*[*elt-2p*::nGFP] | wild-type (CX0007) co-injection marker control |
| 2-S1 | *elt-2p*::nGFP; edited *eat-4* | CX17461 x  CX18118 | *eat-4(ky1091) eat-4(ky1092)* *III; kyEx6458*[*elt-2p*::nGFP] | co-injection marker & *eat-4(ky1091) eat-4(ky1092)* control |
| 2-S2 | *tbh-1* | MT9455 | *tbh-1(n3247)* *X* | backcrossed to N2 8x for autosomes, 4x for *X* |
| 4, 4-S1, 4-S2,  6-S2 | wild-type | N2 | N2 | Animals in Figure 6-S2 are derived from transgene-negative CX14853 |
| 4, 4-S1, 4-S2 | RIM::tetanus toxin | CX14993 | *kyEx4962*[*tdc-1p*::tetanus toxin::mCherry] |  |
| 4-S1 | *tbh-1p*::tetanus toxin | CX17912 | *kyEx6345*[pAS04 = *tbh-1p*::tetanus toxin::mCherry, *unc-1*22*p*::GFP] |  |
| 5, 5-S1,5-S1,5-S2 | RIM::HisCl; wild-type | CX18193 | *kyIs779*[*tdc-1p*::HisCl::SL2::mCherry] | *kyIs779* = apparent spontaneous integrant of *kyEx5464*; not backcrossed parent: CX16040 |
| 5-S1 | RIM::HisCl; *tdc-1* | CX18194 | *tdc-1(n3419)* *II;* *kyIs779*[*tdc-1p*::HisCl::SL2::mCherry] | *tdc-1(n3419)* *II* backcrossed 11x to N2 *kyIs779* = apparent spontaneous integrant of *kyEx5464*; not backcrossed parent: CX16040 |
| 5-S3 | wild-type | CX15380 | *kyEx5170* [*rig-3p*::GCaMP5.0] |  |
| 5-S3 | RIM::HisCl | CX15380 x CX18193 | *kyEx5170* [*rig-3p*::GCaMP5.0]  *kyIs779*[*tdc-1p*::HisCl::SL2::mCherry] | *kyIs779* = apparent spontaneous integrant of *kyEx5464*; not backcrossed parent: CX16040 |
| 6, 4-S1, 6-S1, 6-S3 | wild-type | CX17546 | *kyEx6164*[*unc-1*22*p*::GFP] |  |
| 6, 6-S1, 6-S2 | RIM gap junction KD | CX14853 | *kyEx487*[*tdc-1p*::*unc-1(n494)*::SL2::mCherry, *unc-1*22*p*::GFP] |  |
| 6-S2 | *tdc-1* | MT13113 | *tdc-1(n3419)* *II* |  |
| 6-S2 | RIM gap junction KD; *tdc-1* | CX14853 x MT13113 | *tdc-1(n3419)* *II; kyEx4871*[*tdc-1p*::*unc-1(n494)*::SL2::mCherry, *unc-1*22*p*::GFP] |  |
| 6-S3 | RIC gap junction KD | CX18293 | *kyEx6565*[pAS06 = *tbh-1p::unc-1(n494)* cdna (dominant negative allele)::SL2::mCherry; *unc-1*22*p*::GFP] | parent: CX0007 |
| 7 | RIM::HisCl; RIM gap junction KD | CX18137 | *kyIs779*[*tdc-1p*::HisCl::SL2::mCherry]; *kyEx6465*[pAS05 = *tdc-1p*::*unc-1(n494)*::SL2::GFP, *elt-2p*::mCherry] | *kyIs779* = apparent spontaneous integrant of *kyEx5464;* not backcrossed parents: CX16040 and CX0007 |
| 7, 7-S1 | RIM::ReaChR: wild-type | CX17885 | *kyEx6329*[*tdc-1p*::nFLP, *elt-2p*::nGFP]; *kyEx6232*[*tdc-1p*::ReaChR::SL2::GFP, *myo-3p*::mCherry] |  |
| 7, 7-S1 | RIM::ReaChR: RIM glu KO | CX17886 | *eat-4(ky1091) eat-4(ky1092)* *III; kyEx6329*[*tdc-1p*::nFLP, *elt-2p*::nGFP]; *kyEx6232*[*tdc-1p*::ReaChR::SL2::GFP, *myo-3p*::mCherry] |  |
| 7, 7-S1 | RIM::ReaChR: *tdc-1* | CX17887 | *tdc-1(n3419) II;*  *kyEx6329*[*tdc-1p*::nFLP, *elt-2p*::nGFP]; *kyEx6232*[*tdc-1p*::ReaChR::SL2::GFP, *myo-3p*::mCherry] | *tdc-1(n3419)* *II* backcrossed 11x to N2 |
| 7, 7-S1 | RIM::ReaChR: RIM glu KO; *tdc-1* | CX17888 | *tdc-1(n3419)* *II;* *eat-4(ky1091) eat-4(ky1092)* *III; kyEx6329*[*tdc-1p*::nFLP, *elt-2p*::nGFP] *kyEx6232*[*tdc-1p*::ReaChR::SL2::GFP, *myo-3p*::mCherry] | *tdc-1(n3419)* *II* backcrossed 11x to N2 *eat-4*(*ky1091) eat-4*(*ky1092)* backcrossed 3x to N2 See López-Cruz et al., 2019 |
| 7, 7-S1 | RIM::ReaChR: wild-type | CX17694 | *kyEx6232*[*tdc-1p*::ReaChR::SL2::GFP, *myo-3p*::mCherry] |  |
| 7, 7-S1 | RIM::ReaChR: RIM gap junction KD | CX18195 | *kyEx6232*[*tdc-1p*::ReaChR::SL2::GFP, *myo-3p*::mCherry]; *kyEx4871*[*tdc-1p*::*unc-1(n494)*::SL2::mCherry, *unc-1*22*p*::GFP] |  |

**Supplementary File 1b. Table 2. Plasmids Generated for This Study**

| **Name** | **Description** | **Figures** |
| --- | --- | --- |
| pAS03 | *tdc-1p*::nFLP | 1-3, 2-S1, 2-S2 |
| pAS04 | *tbh-1p*::tetanus toxin::mCherry | 4-S1 |
| pAS05 | *tdc-1p*::*unc-1(n494)*::SL2::GFP | 7 |
| pAS06 | *tbh-1p*::*unc-1(n494)*::SL2::mCherry | 6-S3 |

**Supplementary File 1c. Table 3. Reversals and Forward Runs, n values**

| **Figures** | **Strain** | **All Reversals** | **Reversal Omegas** | **Pure Reversals** | **Forward Runs** |
| --- | --- | --- | --- | --- | --- |
| 2-3, 2-S2 | wild-type | 1443 | 605 | 555 | 1898 |
| 2-3, 2-S2 | RIM glu KO | 2689 | 1142 | 1011 | 3121 |
| 2-3, 2-S2 | *tdc-1* | 2760 | 628 | 1462 | 3132 |
| 2-3, 2-S2 | RIM glu KO; *tdc-1* | 2217 | 500 | 1231 | 2594 |
| 2-S1 | *elt-2p*::nGFP; wild-type | 642 |  |  | 861 |
| 2-S1 | *elt-2p*::nGFP; edited *eat-4* | 634 |  |  | 913 |
| 2-S1 | *elt-2p*::nGFP; tdc-1*p*::nFLP; wild-type | 584 |  |  | 687 |
| 2-S2 | wild-type | 532 |  |  | 636 |
| 2-S2 | *tbh-1* | 384 |  |  | 618 |
| 4, 4-S1, 4-S2 | wild-type | 595 | 239 | 211 | 657 |
| 4, 4-S1, 4-S2 | RIM::tetanus toxin | 1066 | 185 | 647 | 1301 |
| 4-S1 | wild-type | 504 |  |  | 664 |
| 4-S1 | tbh-1*p*::tetanus toxin | 733 |  |  | 1041 |
| 5, 5-S1 | RIM::HisCl; wild-type (- his) | 853 |  |  | 1071 |
| 5, 5-S1 | RIM::HisCl; wild-type (+ his) | 394 |  |  | 778 |
| 5 | RIM::HisCl; wild-type (- chronic his) | 389 |  |  | 487 |
| 5 | RIM::HisCl; wild-type (+ chronic his) | 156 |  |  | 303 |
| 5-S1 | RIM::HisCl; *tdc-1* (- his) | 856 |  |  | 1250 |
| 5-S1 | RIM::HisCl; *tdc-1* (+ his) | 358 |  |  | 1096 |
| 6, 6-S1 | wild-type | 330 | 116 | 134 | 355 |
| 6, 6-S1 | RIM gap junction KD | 933 | 193 | 473 | 889 |
| 6-S2 | wild-type | 691 |  |  | 875 |
| 6-S2 | *tdc-1* | 813 |  |  | 1015 |
| 6-S2 | RIM gap junction KD | 1100 |  |  | 1055 |
| 6-S2 | RIM gap junction KD; *tdc-1* | 1432 |  |  | 1541 |
| 6-S3 | wild-type | 599 |  |  | 787 |
| 6-S3 | tbh-1p::gap junction KD | 537 |  |  | 927 |
| 7 | RIM::HisCl; wild-type (- his) | 542 |  |  | 768 |
| 7 | RIM::HisCl; wild-type (+ his) | 261 |  |  | 779 |
| 7 | RIM::HisCl; RIM gap junction KD (- his) | 1723 |  |  | 1994 |
| 7 | RIM::HisCl; RIM gap junction KD (+ his) | 649 |  |  | 1231 |
| 7,7-S1 | RIM::ReaChR; wild-type | 167 |  |  | N/A |
| 7, 7-S1 | RIM::ReaChR; RIM glu KO | 159 |  |  | N/A |
| 7, 7-S1 | RIM::ReaChR; *tdc-1* | 159 |  |  | N/A |
| 7, 7-S1 | RIM::ReaChR; RIM glu KO; *tdc-1* | 193 |  |  | N/A |
| 7, 7-S1 | RIM::ReaChR; wild-type | 150 |  |  | N/A |
| 7, 7-S1 | RIM::ReaChR; RIM gap junction KD | 119 |  |  | N/A |

**Supplementary File 1d. Table 4. Statistical Analyses**

| **Panel** | **Comparison** | **Summary** | **p-value** | **alpha** | **Test** |
| --- | --- | --- | --- | --- | --- |
| 2B; left | wild-type vs RIM glu KO | *** | <0.0001 | 0.05 | Kruskal-Wallis; Dunn's correction |
| 2B; left | wild-type vs *tdc-1* | *** | <0.0001 | 0.05 | Kruskal-Wallis; Dunn's correction |
| 2B; left | wild-type vs RIM glu KO; *tdc-1* | *** | 0.0050 | 0.05 | Kruskal-Wallis; Dunn's correction |
| 2B; right | wild-type vs RIM glu KO | ns | >0.9999 | 0.05 | Kruskal-Wallis; Dunn's correction |
| 2B; right | wild-type vs *tdc-1* | ns | 0.4410 | 0.05 | Kruskal-Wallis; Dunn's correction |
| 2B; right | wild-type vs RIM glu KO; *tdc-1* | ns | 0.0614 | 0.05 | Kruskal-Wallis; Dunn's correction |
| 2D; left | wild-type vs RIM glu KO | *** | <0.0001 | 0.05 | Kruskal-Wallis; Dunn's correction |
| 2D; left | wild-type vs *tdc-1* | ns | >0.9999 | 0.05 | Kruskal-Wallis; Dunn's correction |
| 2D; left | wild-type vs RIM glu KO; *tdc-1* | ns | >0.9999 | 0.05 | Kruskal-Wallis; Dunn's correction |
| 2D; right | wild-type vs RIM glu KO | *** | 0.0003 | 0.05 | Kruskal-Wallis; Dunn's correction |
| 2D; right | wild-type vs *tdc-1* | *** | <0.0001 | 0.05 | Kruskal-Wallis; Dunn's correction |
| 2D; right | wild-type vs RIM glu KO; *tdc-1* | *** | <0.0001 | 0.05 | Kruskal-Wallis; Dunn's correction |
| 2G; left | wild-type vs RIM glu KO | ns | 0.9869 | 0.05 | Kruskal-Wallis; Dunn's correction |
| 2G; left | wild-type vs *tdc-1* | *** | <0.0001 | 0.05 | Kruskal-Wallis; Dunn's correction |
| 2G; left | wild-type vs RIM glu KO; *tdc-1* | *** | <0.0001 | 0.05 | Kruskal-Wallis; Dunn's correction |
| 2G; right | wild-type vs RIM glu KO | ns | >0.9999 | 0.05 | Kruskal-Wallis; Dunn's correction |
| 2G; right | wild-type vs *tdc-1* | ** | 0.0056 | 0.05 | Kruskal-Wallis; Dunn's correction |
| 2G; right | wild-type vs RIM glu KO; *tdc-1* | *** | <0.0001 | 0.05 | Kruskal-Wallis; Dunn's correction |
| 2H | wild-type vs RIM glu KO | ns | >0.9999 | 0.05 | Kruskal-Wallis; Dunn's correction |
| 2H | wild-type vs *tdc-1* | *** | <0.0001 | 0.05 | Kruskal-Wallis; Dunn's correction |
| 2H | wild-type vs RIM glu KO; *tdc-1* | *** | <0.0001 | 0.05 | Kruskal-Wallis; Dunn's correction |
| 2-S1A | *elt-2p*::nGFP; WT vs *elt-2p*::nGFP; edited *eat-4* | ns | 0.9470 | 0.05 | Mann-Whitney |
| 2-S1A | *elt-2p*::nGFP; WT vs *elt-2p*::nGFP; *tdc-1p*::nFLP; WT | ns | 0.8785 | 0.05 | Mann-Whitney |
| 2-S1B; left | *elt-2p*::nGFP; WT vs *elt-2p*::nGFP; edited *eat-4* | ns | 0.3154 | 0.05 | Mann-Whitney |
| 2-S1B; left | *elt-2p*::nGFP; WT vs *elt-2p*::nGFP; *tdc-1p*::nFLP; WT | ns | 0.6965 | 0.05 | Mann-Whitney |
| 2-S1B; right | *elt-2p*::nGFP; WT vs *elt-2p*::nGFP; edited *eat-4* | ns | 0.2112 | 0.05 | Mann-Whitney |
| 2-S1B; right | *elt-2p*::nGFP; WT vs *elt-2p*::nGFP; *tdc-1p*::nFLP; WT | ns | 0.5726 | 0.05 | Mann-Whitney |
| 2-S1C | *elt-2p*::nGFP; WT vs *elt-2p*::nGFP; edited *eat-4* | ns | 0.8460 | 0.05 | Mann-Whitney |
| 2-S1C | *elt-2p*::nGFP; WT vs *elt-2p*::nGFP; *tdc-1p*::nFLP; WT | ns | 0.9473 | 0.05 | Mann-Whitney |
| 2-S1D | *elt-2p*::nGFP; WT vs *elt-2p*::nGFP; edited *eat-4* | ns | 0.3153 | 0.05 | 2-sample Kolmogorov-Smirnov |
| 2-S1D | *elt-2p*::nGFP; WT vs *elt-2p*::nGFP; *tdc-1p*::nFLP; WT | ** | 0.0023 | 0.05 | 2-sample Kolmogorov-Smirnov |
| 2-S1E | *elt-2p*::nGFP; WT vs *elt-2p*::nGFP; edited *eat-4* | ns | 0.1197 | 0.05 | 2-sample Kolmogorov-Smirnov |
| 2-S1E | *elt-2p*::nGFP; WT vs *elt-2p*::nGFP; *tdc-1p*::nFLP; WT | *** | 0.0002 | 0.05 | 2-sample Kolmogorov-Smirnov |
| 2-S1F | *elt-2p*::nGFP; WT vs *elt-2p*::nGFP; edited *eat-4* | ns | 0.2853 | 0.05 | 2-sample Kolmogorov-Smirnov |
| 2-S1F | *elt-2p*::nGFP; WT vs *elt-2p*::nGFP; *tdc-1p*::nFLP; WT | ** | 0.0057 | 0.05 | 2-sample Kolmogorov-Smirnov |
| 2-S1G | *elt-2p*::nGFP; WT vs *elt-2p*::nGFP; edited *eat-4* | * | 0.0246 | 0.05 | 2-sample Kolmogorov-Smirnov |
| 2-S1G | *elt-2p*::nGFP; WT vs *elt-2p*::nGFP; *tdc-1p*::nFLP; WT | ns | 0.2849 | 0.05 | 2-sample Kolmogorov-Smirnov |
| 2-S1H | *elt-2p*::nGFP; WT vs *elt-2p*::nGFP; edited *eat-4* | ns | 0.2050 | 0.05 | 2-sample Kolmogorov-Smirnov |
| 2-S1H | *elt-2p*::nGFP; WT vs *elt-2p*::nGFP; *tdc-1p*::nFLP; WT | ns | 0.3725 | 0.05 | 2-sample Kolmogorov-Smirnov |
| 2-S2A | wild-type vs *tbh-1* | ns | 0.8763 | 0.05 | Mann-Whitney |
| 2-S2B; left | wild-type vs *tbh-1* | ns | 0.5303 | 0.05 | Mann-Whitney |
| 2-S2B; right | wild-type vs *tbh-1* | ns | 0.2677 | 0.05 | Mann-Whitney |
| 2-S2C | wild-type vs *tbh-1* | * | 0.0101 | 0.05 | Mann-Whitney |
| 2-S2D | wild-type vs *tbh-1* | ** | 0.0046 | 0.05 | 2-sample Kolmogorov-Smirnov |
| 2-S2E | wild-type vs *tbh-1* | *** | <0.0001 | 0.05 | 2-sample Kolmogorov-Smirnov |
| 2-S2F | wild-type vs *tbh-1* | *** | <0.0001 | 0.05 | 2-sample Kolmogorov-Smirnov |
| 2-S2G | wild-type vs *tbh-1* | ** | 0.0019 | 0.05 | 2-sample Kolmogorov-Smirnov |
| 2-S2H | wild-type vs *tbh-1* | *** | <0.0001 | 0.05 | 2-sample Kolmogorov-Smirnov |
| 2-S2J | wild-type vs RIM glu KO | *** | <0.0001 | 0.05 | 2-sample Kolmogorov-Smirnov; Bonferroni correction |
| 2-S2J | wild-type vs *tdc-1* | *** | <0.0001 | 0.05 | 2-sample Kolmogorov-Smirnov; Bonferroni correction |
| 2-S2J | wild-type vs RIM glu KO; *tdc-1* | *** | <0.0001 | 0.05 | 2-sample Kolmogorov-Smirnov; Bonferroni correction |
| 2-S2K | wild-type vs RIM glu KO | *** | <0.0001 | 0.05 | 2-sample Kolmogorov-Smirnov; Bonferroni correction |
| 2-S2K | wild-type vs *tdc-1* | *** | <0.0001 | 0.05 | 2-sample Kolmogorov-Smirnov; Bonferroni correction |
| 2-S2K | wild-type vs RIM glu KO; *tdc-1* | *** | <0.0001 | 0.05 | 2-sample Kolmogorov-Smirnov; Bonferroni correction |
| 2-S2L | wild-type vs RIM glu KO | ns | >0.9999 | 0.05 | Kruskal-Wallis; Dunn's correction |
| 2-S2L | wild-type vs *tdc-1* | *** | 0.0002 | 0.05 | Kruskal-Wallis; Dunn's correction |
| 2-S2L | wild-type vs RIM glu KO; *tdc-1* | *** | <0.0001 | 0.05 | Kruskal-Wallis; Dunn's correction |
| 3A | wild-type vs RIM glu KO | *** | <0.0001 | 0.05 | 2-sample Kolmogorov-Smirnov; Bonferroni correction |
| 3A | wild-type vs *tdc-1* | *** | <0.0001 | 0.05 | 2-sample Kolmogorov-Smirnov; Bonferroni correction |
| 3A | wild-type vs RIM glu KO; *tdc-1* | *** | <0.0001 | 0.05 | 2-sample Kolmogorov-Smirnov; Bonferroni correction |
| 3B | wild-type vs RIM glu KO | *** | <0.0001 | 0.05 | 2-sample Kolmogorov-Smirnov; Bonferroni correction |
| 3B | wild-type vs *tdc-1* | *** | <0.0001 | 0.05 | 2-sample Kolmogorov-Smirnov; Bonferroni correction |
| 3B | wild-type vs RIM glu KO; *tdc-1* | *** | <0.0001 | 0.05 | 2-sample Kolmogorov-Smirnov; Bonferroni correction |
| 3C | wild-type vs RIM glu KO | *** | <0.0001 | 0.05 | 2-sample Kolmogorov-Smirnov; Bonferroni correction |
| 3C | wild-type vs *tdc-1* | *** | <0.0001 | 0.05 | 2-sample Kolmogorov-Smirnov; Bonferroni correction |
| 3C | wild-type vs RIM glu KO; *tdc-1* | *** | <0.0001 | 0.05 | 2-sample Kolmogorov-Smirnov; Bonferroni correction |
| 3D | wild-type vs RIM glu KO | *** | <0.0001 | 0.05 | 2-sample Kolmogorov-Smirnov; Bonferroni correction |
| 3D | wild-type vs *tdc-1* | *** | <0.0001 | 0.05 | 2-sample Kolmogorov-Smirnov; Bonferroni correction |
| 3D | wild-type vs RIM glu KO; *tdc-1* | *** | <0.0001 | 0.05 | 2-sample Kolmogorov-Smirnov; Bonferroni correction |
| 3E | wild-type vs RIM glu KO | *** | <0.0001 | 0.05 | 2-sample Kolmogorov-Smirnov; Bonferroni correction |
| 3E | wild-type vs *tdc-1* | *** | <0.0001 | 0.05 | 2-sample Kolmogorov-Smirnov; Bonferroni correction |
| 3E | wild-type vs RIM glu KO; *tdc-1* | *** | <0.0001 | 0.05 | 2-sample Kolmogorov-Smirnov; Bonferroni correction |
| 3F | wild-type vs RIM glu KO | *** | <0.0001 | 0.05 | 2-sample Kolmogorov-Smirnov; Bonferroni correction |
| 3F | wild-type vs *tdc-1* | *** | <0.0001 | 0.05 | 2-sample Kolmogorov-Smirnov; Bonferroni correction |
| 3F | wild-type vs RIM glu KO; *tdc-1* | *** | <0.0001 | 0.05 | 2-sample Kolmogorov-Smirnov; Bonferroni correction |
| 3G | wild-type vs RIM glu KO | * | 0.0444 | 0.05 | 2-sample Kolmogorov-Smirnov; Bonferroni correction |
| 3G | wild-type vs *tdc-1* | *** | <0.0001 | 0.05 | 2-sample Kolmogorov-Smirnov; Bonferroni correction |
| 3G | wild-type vs RIM glu KO; *tdc-1* | *** | <0.0001 | 0.05 | 2-sample Kolmogorov-Smirnov; Bonferroni correction |
| 3H | wild-type vs RIM glu KO | ns | 0.2622 | 0.05 | 2-sample Kolmogorov-Smirnov; Bonferroni correction |
| 3H | wild-type vs *tdc-1* | *** | <0.0001 | 0.05 | 2-sample Kolmogorov-Smirnov; Bonferroni correction |
| 3H | wild-type vs RIM glu KO; *tdc-1* | *** | <0.0001 | 0.05 | 2-sample Kolmogorov-Smirnov; Bonferroni correction |
| 3I | wild-type vs RIM glu KO | ns | 0.8643 | 0.05 | 2-sample Kolmogorov-Smirnov; Bonferroni correction |
| 3I | wild-type vs *tdc-1* | *** | <0.0001 | 0.05 | 2-sample Kolmogorov-Smirnov; Bonferroni correction |
| 3I | wild-type vs RIM glu KO; *tdc-1* | *** | <0.0001 | 0.05 | 2-sample Kolmogorov-Smirnov; Bonferroni correction |
| 4C; left | wild-type vs RIM::tetanus toxin | ** | 0.0070 | 0.05 | Mann-Whitney |
| 4C; right | wild-type vs RIM::tetanus toxin | * | 0.0281 | 0.05 | Mann-Whitney |
| 4D; left | wild-type vs RIM::tetanus toxin | ns | 0.1105 | 0.05 | Mann-Whitney |
| 4D; right | wild-type vs RIM::tetanus toxin | *** | 0.0002 | 0.05 | Mann-Whitney |
| 4E | wild-type vs RIM::tetanus toxin | *** | <0.0001 | 0.05 | 2-sample Kolmogorov-Smirnov |
| 4F | wild-type vs RIM::tetanus toxin | *** | <0.0001 | 0.05 | 2-sample Kolmogorov-Smirnov |
| 4G | wild-type vs RIM::tetanus toxin | *** | <0.0001 | 0.05 | 2-sample Kolmogorov-Smirnov |
| 4-S1A; left | wild-type vs *tbh-1p*::tetanus toxin | *** | 0.0006 | 0.05 | Mann-Whitney |
| 4-S1A; right | wild-type vs *tbh-1p*::tetanus toxin | ns | 0.1304 | 0.05 | Mann-Whitney |
| 4-S1B; left | wild-type vs *tbh-1p*::tetanus toxin | ** | 0.0022 | 0.05 | Mann-Whitney |
| 4-S1B; right | wild-type vs *tbh-1p*::tetanus toxin | * | 0.0207 | 0.05 | Mann-Whitney |
| 4-S1C | wild-type vs *tbh-1p*::tetanus toxin | ns | 0.6943 | 0.05 | 2-sample Kolmogorov-Smirnov |
| 4-S1D | wild-type vs *tbh-1p*::tetanus toxin | *** | <0.0001 | 0.05 | 2-sample Kolmogorov-Smirnov |
| 4-S1E | wild-type vs *tbh-1p*::tetanus toxin | ** | 0.0055 | 0.05 | 2-sample Kolmogorov-Smirnov |
| 4-S1F | wild-type vs *tbh-1p*::tetanus toxin | *** | <0.0001 | 0.05 | 2-sample Kolmogorov-Smirnov |
| 4-S1G | wild-type vs *tbh-1p*::tetanus toxin | *** | <0.0001 | 0.05 | 2-sample Kolmogorov-Smirnov |
| 4-S1H | wild-type vs *tbh-1p*::tetanus toxin | * | 0.0101 | 0.05 | Mann-Whitney |
| 4-S1I | wild-type vs RIM::tetanus toxin | *** | <0.0001 | 0.05 | 2-sample Kolmogorov-Smirnov |
| 4-S1J | wild-type vs RIM::tetanus toxin | *** | <0.0001 | 0.05 | 2-sample Kolmogorov-Smirnov |
| 4-S1K | wild-type vs RIM::tetanus toxin | ** | 0.0019 | 0.05 | Mann-Whitney |
| 4-S2A; left | wild-type vs RIM::tetanus toxin | *** | 0.0002 | 0.05 | Mann-Whitney |
| 4-S2A; right | wild-type vs RIM::tetanus toxin | *** | 0.0006 | 0.05 | Mann-Whitney |
| 4-S2B; left | wild-type vs RIM::tetanus toxin | *** | <0.0001 | 0.05 | 2-sample Kolmogorov-Smirnov |
| 4-S2B; right | wild-type vs RIM::tetanus toxin | *** | <0.0001 | 0.05 | 2-sample Kolmogorov-Smirnov |
| 5C | RIM::HisCl; wild-type: (- his) vs (+ his) | *** | <0.0001 | 0.05 | Mann-Whitney |
| 5D | RIM::HisCl; wild-type: (- his) vs (+ his) | *** | <0.0001 | 0.05 | 2-sample Kolmogorov-Smirnov |
| 5E; left | RIM::HisCl; wild-type: (- his) vs (+ his) | *** | <0.0001 | 0.05 | 2-sample Kolmogorov-Smirnov |
| 5E; right | RIM::HisCl; wild-type: (- his) vs (+ his) | *** | <0.0001 | 0.05 | 2-sample Kolmogorov-Smirnov |
| 5E; center | RIM::HisCl; wild-type: (- his) vs (+ his) | *** | <0.0001 | 0.05 | 2-sample Kolmogorov-Smirnov |
| 5F | RIM::HisCl; wild-type: (-chronic his) vs (+ chronic his) | *** | 0.0002 | 0.05 | Mann-Whitney |
| 5G | RIM::HisCl; wild-type: (-chronic his) vs (+ chronic his) | ** | 0.0012 | 0.05 | 2-sample Kolmogorov-Smirnov |
| 5H; left | RIM::HisCl;wild-type: (-chronic his) vs (+ chronic his) | *** | <0.0001 | 0.05 | 2-sample Kolmogorov-Smirnov |
| 5H; center | RIM::HisCl; wild-type: (-chronic his) vs (+ chronic his) | *** | <0.0001 | 0.05 | 2-sample Kolmogorov-Smirnov |
| 5H; right | RIM::HisCl; wild-type: (-chronic his) vs (+ chronic his) | ** | 0.002 | 0.05 | 2-sample Kolmogorov-Smirnov |
| 5S-1A | RIM::HisCl; wild-type: (- his) vs (+ his) | *** | 0.0002 | 0.05 | Kruskal-Wallis; Dunn's correction |
| 5S-1A | RIM::HisCl; wild-type (- his) vs RIM::HisCl; *tdc-1* (- his) | ns | 0.613 | 0.05 | Kruskal-Wallis; Dunn's correction |
| 5S-1A | RIM::HisCl; wild-type (- his) vs RIM::HisCl; *tdc-1* (+ his) | *** | <0.0001 | 0.05 | Kruskal-Wallis; Dunn's correction |
| 5S-1B | RIM::HisCl; wild-type: (- his) vs (+ his) | *** | <0.0001 | 0.05 | 2-sample Kolmogorov-Smirnov; Bonferroni correction |
| 5S-1B | RIM::HisCl; wild-type (- his) vs RIM::HisCl; *tdc-1* (- his) | * | 0.0219 | 0.05 | 2-sample Kolmogorov-Smirnov; Bonferroni correction |
| 5S-1B | RIM::HisCl; wild-type (- his) vs RIM::HisCl; *tdc-1* (+ his) | *** | <0.0001 | 0.05 | 2-sample Kolmogorov-Smirnov; Bonferroni correction |
| 5S-1C | RIM::HisCl; wild-type: (- his) vs (+ his) | *** | <0.0001 | 0.05 | 2-sample Kolmogorov-Smirnov; Bonferroni correction |
| 5S-1C | RIM::HisCl; wild-type (- his) vs RIM::HisCl; *tdc-1* (- his) | *** | <0.0001 | 0.05 | 2-sample Kolmogorov-Smirnov; Bonferroni correction |
| 5S-1C | RIM::HisCl; wild-type (- his) vs RIM::HisCl; *tdc-1* (+ his) | *** | <0.0001 | 0.05 | 2-sample Kolmogorov-Smirnov; Bonferroni correction |
| 5S-1D | RIM::HisCl; wild-type: (- his) vs (+ his) | *** | <0.0001 | 0.05 | 2-sample Kolmogorov-Smirnov; Bonferroni correction |
| 5S-1D | RIM::HisCl; wild-type (- his) vs RIM::HisCl; *tdc-1* (- his) | *** | <0.0001 | 0.05 | 2-sample Kolmogorov-Smirnov; Bonferroni correction |
| 5S-1D | RIM::HisCl; wild-type (- his) vs RIM::HisCl; *tdc-1* (+ his) | *** | <0.0001 | 0.05 | 2-sample Kolmogorov-Smirnov; Bonferroni correction |
| 5S-1E | RIM::HisCl; wild-type: (- his) vs (+ his) | *** | <0.0001 | 0.05 | 2-sample Kolmogorov-Smirnov; Bonferroni correction |
| 5S-1E | RIM::HisCl; wild-type (- his) vs RIM::HisCl; *tdc-1* (- his) | *** | <0.0001 | 0.05 | 2-sample Kolmogorov-Smirnov; Bonferroni correction |
| 5S-1E | RIM::HisCl; wild-type (- his) vs RIM::HisCl; *tdc-1* (+ his) | *** | <0.0001 | 0.05 | 2-sample Kolmogorov-Smirnov; Bonferroni correction |
| 5-S1B; left | RIM::HisCl; wild-type (- chronic his) vs  RIM::HisCl; wild-type (+chronic his) ; chronic treatment | *** | 0.0002 | 0.05 | Mann-Whitney |
| 5-S1B; right | RIM::HisCl; wild-type (- chronic his) vs  RIM::HisCl; wild-type (+chronic his) ; recovery | ns | >0.9999 | 0.05 | Mann-Whitney |
| 5-S3C; left | wild-type (+ his) vs RIM::HisCl (+ his) | *** | 0.0002 | 0.05 | Mann-Whitney |
| 5-S3C; right | wild-type (+ his) vs RIM::HisCl (+ his) | ns | 0.8129 | 0.05 | Mann-Whitney |
| 5-S3D; left | wild-type (+ his) vs RIM::HisCl (+ his) | *** | <0.0001 | 0.05 | Mann-Whitney |
| 5-S3D; right | wild-type (+ his) vs RIM::HisCl (+ his) | * | 0.0157 | 0.05 | Mann-Whitney |
| 5-S3E; left | wild-type (+ his) vs RIM::HisCl (+ his) | ns | 0.5974 | 0.05 | Mann-Whitney |
| 5-S3E; right | wild-type (+ his) vs RIM::HisCl (+ his) | ns | 0.0646 | 0.05 | Mann-Whitney |
| 6C; left | wild-type vs RIM gap junction KD | ** | 0.0022 | 0.05 | Mann-Whitney |
| 6C; right | wild-type vs RIM gap junction KD | * | 0.0411 | 0.05 | Mann-Whitney |
| 6D; left | wild-type vs RIM gap junction KD | ** | 0.0022 | 0.05 | Mann-Whitney |
| 6D; right | wild-type vs RIM gap junction KD | ** | 0.0022 | 0.05 | Mann-Whitney |
| 6E | wild-type vs RIM gap junction KD | ** | 0.0058 | 0.05 | 2-sample Kolmogorov-Smirnov |
| 6F | wild-type vs RIM gap junction KD | *** | 0.0008 | 0.05 | 2-sample Kolmogorov-Smirnov |
| 6G | wild-type vs RIM gap junction KD | ns | 0.4306 | 0.05 | 2-sample Kolmogorov-Smirnov |
| 6H | wild-type vs RIM gap junction KD | *** | <0.0001 | 0.05 | 2-sample Kolmogorov-Smirnov |
| 6-S1A; left | wild-type vs RIM gap junction KD | ** | 0.0043 | 0.05 | Mann-Whitney |
| 6-S1A; right | wild-type vs RIM gap junction KD | ns | 0.3939 | 0.05 | Mann-Whitney |
| 6-S1B; left | wild-type vs RIM gap junction KD | ns | 0.2682 | 0.05 | 2-sample Kolmogorov-Smirnov |
| 6-S1B; right | wild-type vs RIM gap junction KD | *** | 0.0004 | 0.05 | 2-sample Kolmogorov-Smirnov |
| 6-S1D | wild-type vs RIM gap junction KD | *** | <0.0001 | 0.05 | 2-sample Kolmogorov-Smirnov |
| 6-S1E | wild-type vs RIM gap junction KD | ** | 0.0022 | 0.05 | Mann-Whitney |
| 6-S2A | wild-type vs *tdc-1* | ns | 0.6683 | 0.05 | Kruskal-Wallis; Dunn's correction |
| 6-S2A | wild-type vs RIM gap junction KD | ** | 0.0099 | 0.05 | Kruskal-Wallis; Dunn's correction |
| 6-S2A | wild-type vs *tdc-1*; RIM gap junction KD | *** | <0.0001 | 0.05 | Kruskal-Wallis; Dunn's correction |
| 6-S2B; left | wild-type vs *tdc-1* | ns | 0.5666 | 0.05 | Kruskal-Wallis; Dunn's correction |
| 6-S2B; left | wild-type vs RIM gap junction KD | ** | 0.0052 | 0.05 | Kruskal-Wallis; Dunn's correction |
| 6-S2B; left | wild-type vs *tdc-1*; RIM gap junction KD | ns | 0.7123 | 0.05 | Kruskal-Wallis; Dunn's correction |
| 6-S2B; right | wild-type vs *tdc-1* | ns | 0.0993 | 0.05 | Kruskal-Wallis; Dunn's correction |
| 6-S2B; right | wild-type vs RIM gap junction KD | * | 0.0250 | 0.05 | Kruskal-Wallis; Dunn's correction |
| 6-S2B; right | wild-type vs *tdc-1*; RIM gap junction KD | *** | <0.0001 | 0.05 | Kruskal-Wallis; Dunn's correction |
| 6-S2C | wild-type vs *tdc-1* | *** | <0.0001 | 0.05 | Kruskal-Wallis; Dunn's correction |
| 6-S2C | wild-type vs RIM gap junction KD | ns | 0.5263 | 0.05 | Kruskal-Wallis; Dunn's correction |
| 6-S2C | wild-type vs *tdc-1*; RIM gap junction KD | ** | 0.0025 | 0.05 | Kruskal-Wallis; Dunn's correction |
| 6-S2D | wild-type vs *tdc-1* | *** | <0.0001 | 0.05 | 2-sample Kolmogorov-Smirnov; Bonferroni correction |
| 6-S2D | wild-type vs RIM gap junction KD | *** | <0.0001 | 0.05 | 2-sample Kolmogorov-Smirnov; Bonferroni correction |
| 6-S2D | wild-type vs *tdc-1*; RIM gap junction KD | *** | <0.0001 | 0.05 | 2-sample Kolmogorov-Smirnov; Bonferroni correction |
| 6-S2E | wild-type vs *tdc-1* | *** | <0.0001 | 0.05 | 2-sample Kolmogorov-Smirnov; Bonferroni correction |
| 6-S2E | wild-type vs RIM gap junction KD | *** | <0.0001 | 0.05 | 2-sample Kolmogorov-Smirnov; Bonferroni correction |
| 6-S2E | wild-type vs *tdc-1*; RIM gap junction KD | *** | <0.0001 | 0.05 | 2-sample Kolmogorov-Smirnov; Bonferroni correction |
| 6-S2F | wild-type vs *tdc-1* | *** | <0.0001 | 0.05 | 2-sample Kolmogorov-Smirnov; Bonferroni correction |
| 6-S2F | wild-type vs RIM gap junction KD | *** | <0.0001 | 0.05 | 2-sample Kolmogorov-Smirnov; Bonferroni correction |
| 6-S2F | wild-type vs *tdc-1*; RIM gap junction KD | *** | <0.0001 | 0.05 | 2-sample Kolmogorov-Smirnov; Bonferroni correction |
| 6-S2G | wild-type vs *tdc-1* | ns | 0.3591 | 0.05 | 2-sample Kolmogorov-Smirnov; Bonferroni correction |
| 6-S2G | wild-type vs RIM gap junction KD | *** | <0.0001 | 0.05 | 2-sample Kolmogorov-Smirnov; Bonferroni correction |
| 6-S2G | wild-type vs *tdc-1*; RIM gap junction KD | *** | <0.0001 | 0.05 | 2-sample Kolmogorov-Smirnov; Bonferroni correction |
| 6-S2H | wild-type vs *tdc-1* | *** | <0.0001 | 0.05 | 2-sample Kolmogorov-Smirnov; Bonferroni correction |
| 6-S2H | wild-type vs RIM gap junction KD | *** | <0.0001 | 0.05 | 2-sample Kolmogorov-Smirnov; Bonferroni correction |
| 6-S2H | wild-type vs *tdc-1*; RIM gap junction KD | *** | <0.0001 | 0.05 | 2-sample Kolmogorov-Smirnov; Bonferroni correction |
| 6-S3A; left | wild-type vs *tbh-1p*::tetanus toxin | ns | 0.5350 | 0.05 | Mann-Whitney |
| 6-S3A; right | wild-type vs RIC gap junction KD | ns | 0.6931 | 0.05 | Mann-Whitney |
| 6-S3B; left | wild-type vs RIC gap junction KD | ns | 0.0513 | 0.05 | Mann-Whitney |
| 6-S3B; right | wild-type vs RIC gap junction KD | ns | 0.3176 | 0.05 | Mann-Whitney |
| 6-S3C | wild-type vs RIC gap junction KD | ** | 0.0081 | 0.05 | 2-sample Kolmogorov-Smirnov |
| 6-S3D | wild-type RIC gap junction KD | *** | <0.0001 | 0.05 | 2-sample Kolmogorov-Smirnov |
| 6-S3E | wild-type vs RIC gap junction KD | *** | 0.0001 | 0.05 | 2-sample Kolmogorov-Smirnov |
| 6-S3F | wild-type vs RIC gap junction KD | *** | <0.0001 | 0.05 | 2-sample Kolmogorov-Smirnov |
| 6-S3G | wild-type vs RIC gap junction KD | *** | <0.0001 | 0.05 | 2-sample Kolmogorov-Smirnov |
| 6-S3H | wild-type vs RIC gap junction KD | ns | 0.2086 | 0.05 | Mann-Whitney |
| 7B | RIM::HisCl: wild-type (- his) vs wild-type (+ his) | * | 0.0153 | 0.05 | Kruskal-Wallis; Dunn's correction |
| 7B | RIM::HisCl: wild-type (- his) vs RIM GJ KD (- his) | ** | 0.0018 | 0.05 | Kruskal-Wallis; Dunn's correction |
| 7B | RIM::HisCl: wild-type (- his) vs RIM GJ KD (+ his) | ns | >0.8905 | 0.05 | Kruskal-Wallis; Dunn's correction |
| 7C | RIM::HisCl: wild-type (- his) vs wild-type (+ his) | *** | <0.0001 | 0.05 | 2-sample Kolmogorov-Smirnov; Bonferroni correction |
| 7C | RIM::HisCl: wild-type (- his) vs RIM GJ KD (- his) | *** | <0.0001 | 0.05 | 2-sample Kolmogorov-Smirnov; Bonferroni correction |
| 7C | RIM::HisCl: wild-type (- his) vs RIM GJ KD (+ his) | ns | 0.6260 | 0.05 | 2-sample Kolmogorov-Smirnov; Bonferroni correction |
| 7E | RIM::ReaChR: wild-type vs RIM glu KO | ** | 0.0022 | 0.05 | 2-sample Kolmogorov-Smirnov; Bonferroni correction |
| 7E | RIM::ReaChR: wild-type vs *tdc-1* | *** | 0.0002 | 0.05 | 2-sample Kolmogorov-Smirnov; Bonferroni correction |
| 7E | RIM::ReaChR: wild-type vs RIM glu KO; *tdc-1* | *** | <0.0001 | 0.05 | 2-sample Kolmogorov-Smirnov; Bonferroni correction |
| 7G | RIM::ReaChR: wild-type vs RIM gap junction KD | *** | <0.0001 | 0.05 | 2-sample Kolmogorov-Smirnov |
| 7-S1B; left | RIM::HisCl: wild-type (- his) vs wild-type (+ his) | *** | <0.0001 | 0.05 | 2-sample Kolmogorov-Smirnov; Bonferroni correction |
| 7-S1B; left | RIM::HisCl: wild-type (- his) vs *tdc-1* (- his) | *** | <0.0001 | 0.05 | 2-sample Kolmogorov-Smirnov; Bonferroni correction |
| 7-S1B; left | RIM::HisCl: wild-type (- his) vs *tdc-1* (+ his) | *** | <0.0001 | 0.05 | 2-sample Kolmogorov-Smirnov; Bonferroni correction |
| 7-S1B; right | RIM::HisCl: wild-type (- his) vs wild-type (+ his) | ** | 0.0026 | 0.05 | 2-sample Kolmogorov-Smirnov; Bonferroni correction |
| 7-S1B; right | RIM::HisCl: wild-type (- his) vs *tdc-1* (- his) | ns | 0.2085 | 0.05 | 2-sample Kolmogorov-Smirnov; Bonferroni correction |
| 7-S1B; right | RIM::HisCl: wild-type (- his) vs *tdc-1* (+ his) | ns | 0.1635 | 0.05 | 2-sample Kolmogorov-Smirnov; Bonferroni correction |
| 7-S1D; left | RIM::HisCl: wild-type (- his) vs wild-type (+ his) | ** | 0.0015 | 0.05 | 2-sample Kolmogorov-Smirnov; Bonferroni correction |
| 7-S1D; left | RIM::HisCl: wild-type (- his) vs RIM GJ KD (- his) | ns | 0.0897 | 0.05 | 2-sample Kolmogorov-Smirnov; Bonferroni correction |
| 7-S1D; left | RIM::HisCl: wild-type (- his) vs RIM GJ KD (+ his) | *** | 0.0001 | 0.05 | 2-sample Kolmogorov-Smirnov; Bonferroni correction |
| 7-S1D; right | RIM::HisCl: wild-type (- his) vs wild-type (+ his) | * | 0.0102 | 0.05 | 2-sample Kolmogorov-Smirnov; Bonferroni correction |
| 7-S1D; right | RIM::HisCl: wild-type (- his) vs RIM GJ KD (- his) | ns | 0.1463 | 0.05 | 2-sample Kolmogorov-Smirnov; Bonferroni correction |
| 7-S1D; right | RIM::HisCl: wild-type (- his) vs RIM GJ KD (+ his) | ns | >0.9999 | 0.05 | 2-sample Kolmogorov-Smirnov; Bonferroni correction |
| 7-S2A; left | RIM::ReaChR: wild-type vs RIM glu KO | ns | >0.9999 | 0.05 | Kruskal-Wallis; Dunn's correction |
| 7-S2A; left | RIM::ReaChR: wild-type vs *tdc-1* | ns | >0.9999 | 0.05 | Kruskal-Wallis; Dunn's correction |
| 7-S2A; left | RIM::ReaChR: wild-type vs RIM glu KO; *tdc-1* | ns | >0.9999 | 0.05 | Kruskal-Wallis; Dunn's correction |
| 7-S2A; right | RIM::ReaChR: wild-type vs RIM glu KO | ns | 0.7543 | 0.05 | Kruskal-Wallis; Dunn's correction |
| 7-S2A; right | RIM::ReaChR: wild-type vs *tdc-1* | ns | >0.9999 | 0.05 | Kruskal-Wallis; Dunn's correction |
| 7-S2A; right | RIM::ReaChR: wild-type vs RIM glu KO; *tdc-1* | ns | 0.7671 | 0.05 | Kruskal-Wallis; Dunn's correction |
| 7-S2B | RIM::ReaChR: wild-type vs RIM glu KO | *** | <0.0001 | 0.05 | 2-sample Kolmogorov-Smirnov; Bonferroni correction |
| 7-S2B | RIM::ReaChR: wild-type vs *tdc-1* | *** | <0.0001 | 0.05 | 2-sample Kolmogorov-Smirnov; Bonferroni correction |
| 7-S2B | RIM::ReaChR: wild-type vs RIM glu KO; *tdc-1* | *** | <0.0001 | 0.05 | 2-sample Kolmogorov-Smirnov; Bonferroni correction |
| 7-S2C | RIM::ReaChR: wild-type vs RIM glu KO | ns | 0.1260 | 0.05 | 2-sample Kolmogorov-Smirnov; Bonferroni correction |
| 7-S2C | RIM::ReaChR: wild-type vs *tdc-1* | ns | 0.5967 | 0.05 | 2-sample Kolmogorov-Smirnov; Bonferroni correction |
| 7-S2C | RIM::ReaChR: wild-type vs RIM glu KO; *tdc-1* | ns | 0.0501 | 0.05 | 2-sample Kolmogorov-Smirnov; Bonferroni correction |
| 7-S2D; left | RIM::ReaChR: wild-type vs RIM gap junction KD | ns | 0.5512 | 0.05 | Mann-Whitney |
| 7-S2D; right | RIM::ReaChR: wild-type vs RIM gap junction KD | *** | 0.0005 | 0.05 | Mann-Whitney |
| 7-S2E | RIM::ReaChR: wild-type vs RIM gap junction KD | *** | <0.0001 | 0.05 | 2-sample Kolmogorov-Smirnov |
| 7-S2F | RIM::ReaChR: wild-type vs RIM gap junction KD | *** | 0.0002 | 0.05 | 2-sample Kolmogorov-Smirnov |
