# Supplementary material for: Behavioral control by depolarized and hyperpolarized states of an integrating neuron
Source: eLife. 2021 Nov 5;10:e67723. doi: 10.7554/eLife.67723 (PMC8570696; doi:10.7554/eLife.67723)
Supplement: Transparent reporting form [file elife-67723-transrepform1.docx]

***eLife’s* transparent reporting form**

We encourage authors to provide detailed information *within their submission* to facilitate the interpretation and replication of experiments. Authors can upload supporting documentation to indicate the use of appropriate reporting guidelines for health-related research (see [EQUATOR Network](http://www.equator-network.org/%20)), life science research (see the [BioSharing Information Resource](https://biosharing.org/)), or the [ARRIVE guidelines](http://www.plosbiology.org/article/info:doi/10.1371/journal.pbio.1000412) for reporting work involving animal research. Where applicable, authors should refer to any relevant reporting standards documents in this form.

If you have any questions, please consult our Journal Policies and/or contact us: [editorial@elifesciences.org](mailto:editorial@elifesciences.org).

**Sample-size estimation**

- You should state whether an appropriate sample size was computed when the study was being designed
- You should state the statistical method of sample size computation and any required assumptions
- If no explicit power analysis was used, you should describe how you decided what sample (replicate) size (number) to use

Please outline where this information can be found within the submission (e.g., sections or figure legends), or explain why this information doesn’t apply to your submission:

We chose sample sizes based on previous experiments that used the same assays and similar genetic perturbations in López-Cruz et al., 2019, as described in Materials and methods. Experiments were able to detect effect sizes of 0.1 (see figures) but to focus on biologically meaningful results, we discuss only effect sizes ≥0.15.

**Replicates**

- You should report how often each experiment was performed
- You should include a definition of biological versus technical replication
- The data obtained should be provided and sufficient information should be provided to indicate the number of independent biological and/or technical replicates
- If you encountered any outliers, you should describe how these were handled
- Criteria for exclusion/inclusion of data should be clearly stated
- High-throughput sequence data should be uploaded before submission, with a private link for reviewers provided (these are available from both GEO and ArrayExpress)

Please outline where this information can be found within the submission (e.g., sections or figure legends), or explain why this information doesn’t apply to your submission:

The number of assays and animals per assay is included within the figure legends. Assays were replicated in parallel with the appropriate controls over at least two days to account for day-to-day variability as explained in Materials and methods. For optogenetic experiments, the number of light pulses given during each assay is described in the figure legends and Materials and methods.

**Statistical reporting**

- Statistical analysis methods should be described and justified
- Raw data should be presented in figures whenever informative to do so (typically when N per group is less than 10)
- For each experiment, you should identify the statistical tests used, exact values of N, definitions of center, methods of multiple test correction, and dispersion and precision measures (e.g., mean, median, SD, SEM, confidence intervals; and, for the major substantive results, a measure of effect size (e.g., Pearson's r, Cohen's d)
- Report exact p-values wherever possible alongside the summary statistics and 95% confidence intervals. These should be reported for all key questions and not only when the p-value is less than 0.05.

Please outline where this information can be found within the submission (e.g., sections or figure legends), or explain why this information doesn’t apply to your submission:

The statistical tests used are included in the figure legends and statistical analyses are described in Materials and methods. Effect sizes of all Kolmogorov-Smirnov tests with p-values < 0.05 are contained within the figures. Values of n are provided in the figure legends, and exact n values for all distributions are provided in Supplementary file 1c (Table 3). Supplementary file 1d (Table 4) provides p-values and statistical tests organized by figure panel. Numerical values shown in all dot plots are included in the Source data file.

(For large datasets, or papers with a very large number of statistical tests, you may upload a single table file with tests, Ns, etc., with reference to sections in the manuscript.)

**Group allocation**

- Indicate how samples were allocated into experimental groups (in the case of clinical studies, please specify allocation to treatment method); if randomization was used, please also state if restricted randomization was applied
- Indicate if masking was used during group allocation, data collection and/or data analysis

Please outline where this information can be found within the submission (e.g., sections or figure legends), or explain why this information doesn’t apply to your submission:

For reversal frequency analyses animals are grouped by assay plate as described in the figure legends and Materials and methods. All distributions of reversal and forward run parameters pool data for each genotype across assays as described in Materials and methods. Masking was not used.

**Additional data files (“source data”)**

- We encourage you to upload relevant additional data files, such as numerical data that are represented as a graph in a figure, or as a summary table
- Where provided, these should be in the most useful format, and they can be uploaded as “Source data” files linked to a main figure or table
- Include model definition files including the full list of parameters used
- Include code used for data analysis (e.g., R, MatLab)
- Avoid stating that data files are “available upon request”

Please indicate the figures or tables for which source data files have been provided:

All primary behavioral data and relevant functions pertaining to data analysis are available at Dryad (<https://doi.org/10.5061/dryad.ht76hdrf6>) and Github (<https://github.com/BargmannLab/SordilloBargmann2021>). The Source data file includes the summarized data for all dot plots in Figures 2-7. Relevant MatLab code for all analysis of spontaneous behavior can be found at https://github.com/navinpokala/BargmannWormTracker.
